# Addition of Audiovisual Feedback During Standard Compressions Is Associated with Improved Ability

**DOI:** 10.5811/westjem.2017.11.34327

**Published:** 2018-02-26

**Authors:** Steve A. Aguilar, Nicholas Asakawa, Cameron Saffer, Christine Williams, Steven Chuh, Lewei Duan

**Affiliations:** Kaiser Permanente Medical Center, San Diego, Emergency Medicine, San Diego, California

## Abstract

**Introduction:**

A benefit of in-hospital cardiac arrest is the opportunity for rapid initiation of “high-quality” chest compressions as defined by current American Heart Association (AHA) adult guidelines as a depth 2–2.4 inches, full chest recoil, rate 100–120 per minute, and minimal interruptions with a chest compression fraction (CCF) ≥ 60%. The goal of this study was to assess the effect of audiovisual feedback on the ability to maintain high-quality chest compressions as per 2015 updated guidelines.

**Methods:**

Ninety-eight participants were randomized into four groups. Participants were randomly assigned to perform chest compressions with or without use of audiovisual feedback (+/− AVF). Participants were further assigned to perform either standard compressions with a ventilation ratio of 30:2 to simulate cardiopulmonary resuscitation (CPR) without an advanced airway or continuous chest compressions to simulate CPR with an advanced airway. The primary outcome measured was ability to maintain high-quality chest compressions as defined by current 2015 AHA guidelines.

**Results:**

Overall comparisons between continuous and standard chest compressions (n=98) were without significant differences in chest compression dynamics (p’s >0.05). Overall comparisons between +/− AVF (n = 98) were significant for differences in average rate of compressions per minute (p= 0.0241) and proportion of chest compressions within guideline rate recommendations (p = 0.0084). There was a significant difference in the proportion of high quality-chest compressions favoring AVF (p = 0.0399). Comparisons between chest compression strategy groups +/− AVF were significant for differences in compression dynamics favoring AVF (p’s < 0.05).

**Conclusion:**

Overall, AVF is associated with greater ability to maintain high-quality chest compressions per most-recent AHA guidelines. Specifically, AVF was associated with a greater proportion of compressions within ideal rate with standard chest compressions while demonstrating a greater proportion of compressions with simultaneous ideal rate and depth with a continuous compression strategy.

## INTRODUCTION

Despite advances in the field of resuscitation science and modest improvement in outcomes, mortality from in-hospital cardiopulmonary arrest (CPA) remains relatively high.^1^,^2^ However, a common denominator in recent reports of modest outcome improvements in CPA resuscitation has been the link to quality of cardiopulmonary resuscitation (CPR).^3^,^4^ In particular, high-quality chest compressions have been described as the foundation that all additional, “downstream” resuscitative efforts are built upon and highly associated with improved survival and favorable neurological outcomes.^3^–^5^ Most recently, high-quality chest compressions have been defined by the updated 2015 American Heart Association (AHA) adult guidelines as a depth of 2–2.4 in, full chest recoil, a rate between 100–120 beats per minute, and a chest compression fraction (CCF) of at least 60%.^3^

Even when delivered according to guidelines, external manual chest compressions are inherently inefficient, providing only 30% to 40% of normal blood flow to the brain and less than one third of normal blood flow to the heart.^6^–^10^ This inefficiency highlights the need for rescuers to deliver the highest-quality chest compressions in a timely and consistent manner. 11,12

Although the relationship between high-quality chest compressions and improved survival has been well described, concern remains with the reports of trained rescuers performing suboptimal compression depth, rate, and hands-off fraction time (i.e., CFF).^13^–^17^ Rescuer overestimation of depth and underestimation of rate, as well as increased performance fatigue in prolonged situations, may be primary forces in the relatively poor adherence to current guidelines.^18^–^21^ Real-time, CPR performer feedback via defibrillator has been a relatively recent approach in maintaining chest compression performance and associated with continuous high-quality chest compression.^13^,^22^–^26^ Currently, there are no studies investigating the ability to maintain high-quality chest compressions within the current 2015 AHA guidelines with and without the influence of real-time audiovisual feedback (AVF), which may assist in maintaining high-quality chest compression. The goal of this study was to assess the ability to maintain high-quality chest compressions by 2015 updated guidelines both with and without (+/−) AVF in a simulated arrest scenario.

## METHODS

This was a randomized, prospective, observational study conducted within a community hospital with over 22,000 annual inpatient admissions. All participants were voluntary emergency department (ED) and medical-surgery nursing staff with both Basic and Advanced Cardiac Life Support (BLS/ACLS) certification. We obtained institutional review board approval, and written consent was required prior to participation. We defined CPR providers as a two-person team consisting of one participant performing chest compressions while the second administered ventilations via bag-valve mask (BVM). Chest compressions and ventilations were performed on a *Little Anne CPR Training Manikin* (Laerdal Medical, Stavanger, Norway). AVF on chest compression rate and depth was provided to participants through ZOLL See-Thru CPR^®^ on R Series® defibrillators (Zoll Medical Corporation, Chelmsford, USA).

**Population Health Research Capsule**

What do we already know about this issue?

Recent AHA guideline updates call for an upper limit on chest compression rate and depth. Audiovisual feedback (AVF) has been previously associated with improved compliance to previous guidelines.

What was the research question?

Does addition of AVF improve compliance to 2015 updated AHA guidelines?

What was the major finding of the study?

AVF is associated with the ability to maintain high-quality chest compressions as per most-recent AHA guidelines.

How does this improve population health?

Ability to maintain high-quality chest compressions over a greater proportion of time is an important link in the “chain of survival” for cardiac arrest victims.

In a “mock code” scenario, 98 teams were randomly assigned to perform CPR +/− AVF chest compression feedback. Participants were further randomly assigned to perform either standard chest compressions (SC) with a compression-to-ventilation ratio of 30:2 to simulate CPR without an advanced airway or continuous chest compressions (CCC) to simulate CPR with an advanced airway for a total of four distinct groups.^3^ Chest compressions were performed for two minutes, representing a standard cycle interposed between rhythm/pulse checks and/or compressor switch. Defibrillator data for analysis included chest compression rate, depth, and compression fraction over the entire two minutes. The primary outcome measured was ability to maintain high-quality chest compressions as defined by current 2015 AHA guidelines.^3^ Secondary outcomes included group differences in chest compression depth, rate, and fraction time. Based on recent findings per Wutzler et al. on the ability to maintain effective chest compressions we estimated a sample size of at least 68 teams to maintain a two-sided alpha of 0.05, and a power of 80%.^27^ Data are presented as means and standard deviations. We compared CPR variables (depth, rate, compression fraction and ventilations) between respective groups by Mann-Whitney U test or continuous variables and by chi-squared test for categorical variables. Only participants with technically adequate data available were used in this comparison. We considered p values < 0.05 statistically significant. No participants were excluded.

## RESULTS

Overall comparisons between continuous and SC compressions (n=98) were without significant differences in chest compression dynamics (p’s >0.05) (Table 1). Overall comparisons between no AVF and AVF (n = 98) were significant for differences in average rate of compressions per minute (p= 0.0241) as well as proportion of chest compressions within guideline rate recommendations, 37.9% vs. 65%, respectively (p = 0.0084) (Table 2). Finally, there was a significant difference in the proportion of chest compressions simultaneously within current rate and depth guideline recommendations (p = 0.0401). This significant difference in average time of ideal chest compressions favored the AVF cohort (p = 0.0399) (Table 2). All groups were able to maintain CCF ≥80%.

We made comparisons between chest compression strategy groups with the use of AVF (n = 40). With the assistance of AVF, there was a significant difference between the standard and continuous compression groups in average depth, 2.8 (0.38) inches vs. 2.3 (0.62) inches, respectively (p =0.0045). There was a significant difference in the proportion of chest compressions within current guideline- recommended depth (p= 0.0384) (Table 1). Comparisons between chest compression strategy groups without AVF (n =58) were significant for a difference in CCF, though both were at or above current recommendations. Otherwise, comparisons did not yield any significant chest compression dynamic differences between groups (p’s > 0.05) (Table 1).

Within the CCC-only cohort (n=50) there were no significant isolated average compression rate or depth differences between +/− AVF (p’s > 0.05). However, a statistically significant difference was noted between +/− AVF groups and the proportion of compressions within ideal depth, 45% vs. 16.7%, respectively(p = 0.0288) (Table 2). Additionally, within this cohort there was a significant difference between feedback groups and the proportion of individuals with an average rate and depth within current guidelines (p = 0.0209). Subsequent analysis revealed that AVF participants demonstrated a greater proportion of time in high-quality chest compressions as previously defined (p = 0.0259) (Table 2). Finally, we compared the SC compression cohort +/− AVF (n =48). Comparisons were significant for differences in average compression rate between the AVF and no AVF groups, 110 (11.24) per minute vs. 117.8 (12.21) per minute respectively (p = 0.0208) Notably, both are within current guidelines. Additionally, there was a significant difference between groups and the proportion of chest compressions with an average rate within current guidelines (p= 0.0034). (Table 2)

## DISCUSSION

Previous iterations of the AHA’s CPR and Emergency Cardiovascular Care guidelines have recommended chest compression rate ≥ 100 compression/min; however, the 2015 updates have called for a chest compression-rate upper limit of 120/min.^28^ The recommendation appears to be based on both animal studies as well as recent clinical observations from large out-of-hospital cardiac arrest registries describing an association between chest compression rates, return of spontaneous circulation (ROSC), and survival to hospital discharge.^29^–^31^ This makes sense as observations in animal studies have described anterograde coronary blood flow as positively correlated with diastolic aortic pressures and subsequently compression rate.

However, at rates greater than 120 compressions/min, this relationship weakens as diastolic coronary perfusion time decreases.^29^ Regarding human data, recent observations from the Resuscitation Outcomes Consortium registry suggest an optimum target of between 100 and 120 compressions per minute.^29^–^31^ In this randomized, controlled study we report that overall, AVF is associated with a greater ability to provide simultaneously guideline-recommended rate and depth. This is important as previous studies have focused on the proportion of correct chest compression rate and depth; however, it has been shown that despite adequate individual mean values, the actual proportion of chest compressions that fell within guideline criteria simultaneously for rate and depth was low.^3^,^32^ Overall comparisons between SC and CCC cohorts were without significant differences in compression dynamics.

AVF appeared to have an effect regardless of chest compression strategy, with isolated analysis of both compression strategy groups notable for differences. Within the SC group, significant differences were noted in both average rate and proportion of compressions within current guideline recommendations. Analysis of the CCC cohort was notable for the association with AVF, a greater proportion of compression depth within current guidelines and proportion of time with ideal compressions. One potential explanation for the association between AVF and ability to perform “high-quality” chest compressions on a more consistent basis is the ability to possibly avoid early fatigue by “pacing” an individual through the early periods of a highly stressful cardiac arrest situation where one could understandably want to push as fast and hard as possible, which in turn may lead to early fatigue and subsequently “poor quality.”^20^,^33^,^34^ Finally, similar to overall analysis, comparisons between compression strategies without AVF did not result in any significant compression differences.

The isolated effect of AVF on compression dynamic overall appears to be related to compression strategy. Within the CCC cohort, the effect appears to be on ability to maintain ideal depth, while in the SC cohort, the effect appears to be related to rate control. We do note that within this cohort, although a statistically significant difference is noted in average rate of compressions, both are within current guidelines. However, it should be noted that the non-AVF cohort demonstrated an average rate at the most upper level of current recommendations, and more importantly was associated with a lower rate of proportion of compressions with rate within guideline recommendations over the testing period. This is important as recent studies have reported an inverse association between compression rates and depth, with rates above 120/min having the greatest impact on reducing compression depth.^35^–^38^

Recent reports have called this upper rate limit into question and suggest that faster rate limits (120–130/min) may be actually associated with a higher likelihood of ROSC in in-hospital cardiac arrest.^39^ Unfortunately, in that study compression depth was not reported, leaving optimal rates in in-hospital arrest up to continued debate.^39^ Interestingly, within the AVF cohort, chest compression depth appeared to be both deeper on the average and out of guideline-recommended depth for the SC cohort. Yet again, these differences did not translate to overall differences in the proportion of time within recommended depth between compression groups. Chest compression strategy and relationship with AVF may be related to the nature of the strategy. That is, with continuous compressions fatigue may become an issue and feedback on depth may be of greater importance over time while bursts of activity after brief pauses with standard compressions may require greater mindfulness in rate of compressions. Further study into the individual effects of AVF on compression strategy is warranted.

Finally, we note that although the presence of AVF appears to have improved the quality of chest compressions, proportions of high-quality compressions were surprisingly low between all groups with a high of 25% and nadir of 3.3% (Table(s) 1, 2). However, our findings are consistent with reported “effective compressions,” i.e., trial period with mean compression rate and depth within guidelines and CCF ≥80% per Wutzler et al. In their simulation-based study, there was an “effective compression” rate of 25.4% with feedback vs. 12.7% without.^40^ These findings warrant further investigation into possible influencing factors and sources of variation including fatigue, critical care experience, and time since last training update.

## LIMITATIONS

Although we report a significant effect from the addition of AVF, it is difficult to assess how this translates into clinical application, as real-time feedback devices have shown the ability to aid in the delivery of longer effective, steadier chest compressions over time, the outcomes on neurologically intact survival to hospital discharge remain to be seen.^41^ Similarly, we did not account for the potential variability that time from last CPR skills update or years since training may have contributed to our findings. Similarly, there is an inherent limitation with the use of manikins in CPR studies. Manikins have markedly greater stiffness at the onset of compression, and maintain a linear stiffness throughout the usual range of displacement, rather than becoming stiffer with greater chest displacement that is a more human characteristic.^42^,^43^

## CONCLUSION

Overall, audiovisual feedback is associated with greater ability to maintain high-quality chest compressions as defined by most recent AHA guidelines. Specifically, audiovisual feedback was associated with a greater proportion of compressions within ideal rate with standard chest compressions while demonstrating a greater proportion of compressions with simultaneous ideal rate and depth with a continuous compression strategy.

## Figures and Tables

**Table 1:** Cardiopulmonary resuscitation variables of ventilation, chest compression rate, depth, chest compression fraction,and ability to maintain rate and depth as per most current American Heart Association recommendations. Shown as overall, audiovisual feedback, and no audiovisual feedback. Groups compared by chest compression strategy.

	Overall				Audiovisual feedback				No audiovisual feedback			
			
	Continuous chest compression	Standard chest compression	Total	P value	Continuous chest compression	Standard chest compression	Total	P value	Continuous chest compression	Standard chest compression	Total	P value
	(n =50)	(n =48)	(n =98)		(n =20)	(n =20)	(n =40)		(n =30)	(n =28)	(n =58)	
Age (years) (SD)	38.8 (10.64)	36.4 (9.77)	37.7 (10.25)	0.2881	37.4 (11.26)	34.4 (9.84)	35.9 (10.55)	0.481			39 (9.92)	0.486
Gender (male) (%)	52	25	38.8	0.0061*	65	15%	40	0.0012*	43.3	32.1	37.9	0.380
ED RN Staff (%)	90	91.3	90.6	0.3316	90	83.3	86.8	0.3941	90	96.4	93.1	0.334
Average depth (in) (SD)	2.5 (0.69)	2.7 (0.50)	2.6 (0.61)	0.051	2.3 (0.62)	2.8 (0.38)	2.5 (0.55)	0.0045*	2.6 (0.72)	2.7 (0.58)	2.7 (0.65)	0.773
Average rate (per minute) (SD)	119.7 (15.77)	114.5 (12.32)	117.2 (14.35)	0.1130	119 (18.8)	110 (11.24)	114.5 (15.96)	0.1105	120.2 (13.71)	117.8 (12.21)	119 (12.9)	0.596
Compression depth within 2015 guidelines (%)	28	16.7	22.4	0.1789	45	15	30	0.0384*	16.7	17.9	17.2	0.904
Compression rate within 2015 guidelines (%)	48	50	49	0.8431	55	75	65	0.1848	43.3	32.1	37.9	0.380
Chest compression rate and depth simultaneously within 2015 guidelines (%)	12	4.2	8.2	0.1568	25	5	15	0.0765	3.3	3.6	3.4	0.968
Chest compression fraction (%) (SD)	90 (19)	80 (15)	90 (19)	<0.0001*	90 (25)	80 (5)	90 (19)	<0.0001*	100 (12)	80 (20)	90 (19)	<0.0001*
Ventilations (per minute)	9.3 (2.78)	6.2 (1.77)	7.8 (2.8)	<0.0001*	9.5 (2.77)	6.2 (1.77)	7.9 (2.83)	0.0003*	9.2 (2.85)	6.3 (1.81)	7.8 (2.8)	0.007*

*ED*, emergency medicine; *SD*, standard deviation; *RN*, registered nurse.

* Denotes statistical significance.

**Table 2:** Cardiopulmonary resuscitation variables of ventilation, chest compression rate, depth, chest compression fraction, and ability to maintain rate and depth as per most current American Heart Association recommendations. Shown as overall, continuous chest compressions, and standard chest compressions. Groups compared by use of audiovisual feedback.

	Overall				Continuous chest compression				Standard chest compression			
			
	Audiovisual				Audiovisual				Audiovisual			
			
	Yes (n = 40)	No (n = 58)	Total (n = 98)	P value	Yes (n =20)	No (n =30)	Total (n =50)	P value	Yes (n =20)	No (n =28)	Total (n =48)	P value
Age (years) (SD)	35.9 (10.55)	39 (9.92)	37.7 (10.25)	0.0929	37.4 (11.26)	39.8 (10.28)	38.8 (10.64)	0.361	34.4 (9.84)	38 (9.61)	36.4 (9.77)	0.2154
Gender (Male) (%)	40	37.9	38.8	0.8363	65	43.3	52	0.133	15	32	25	0.1763
ED RN staff (%)	86.8	93.1	90.6	0.2202	90	90	90	0.391	83.3	96.4	91.3	0.2384
Average depth (in) (SD)	2.5 (0.55)	2.7 (0.65)	2.6 (0.61)	0.2687	2.3 (0.62)	2.6 (0.72)	2.5 (0.69)	0.867	2.8 (0.38)	2.7 (0.58)	2.7 (0.50)	0.7143
Average rate (per minute) (SD)	114.5 (15.96)	119 (12.95)	117.2 (14.35)	0.0241*	119 (18.8)	120.2 (13.71)	119.7 (15.77)	0.389	110 (11.24)	117.8 (12.21)	114.5 (12.32)	0.0208*
Compression depth within 2015 guidelines (%)	30	17.2	22.4	0.1368	45	16.7	28	0.028*	15	17.9	16.7	0.7934
Compression rate within 2015 guidelines (%)	65	37.9	49	0.0084*	55	43	48	0.418	75	32.1	50	0.0034*
Chest compression rate and depth Simultaneously within 2015 guidelines (%)	15	3.4	8.2	0.0401*	25	3.3	12	0.020*	5%	3.6	4.2	0.8071
Chest compression fraction (%) (SD)	90 (19)	90 (19)	90 (19)	0.9211	90 (25)	100 (12)	90 (19)	0.973	80 (5)	80 (20)	80 (15)	0.5865
Ventilations (per minute) (SD)	7.8 (2.83)	7.8 (2.8)	7.8(2.8)	0.9928	9.5 (2.77)	9.2 (2.85)	9.3 (2.78)	0.951	6.2 (1.77)	6.3 (1.81)	6.2 (1.77)	0.9787

*ED*, emergency medicine; *SD*, standard deviation; *RN*, registered nurse.

* Denotes statistical significance.
